# Curcumin modulates airway remodelling‐contributing genes—the significance of transcription factors

**DOI:** 10.1111/jcmm.17102

**Published:** 2021-12-23

**Authors:** Joanna Wieczfinska, Przemysław Sitarek, Tomasz Kowalczyk, Piotr Rieske, Rafal Pawliczak

**Affiliations:** ^1^ Department of Immunopathology Medical University of Lodz Lodz Poland; ^2^ Department of Biology and Pharmaceutical Botany Medical University of Lodz Lodz Poland; ^3^ Department of Molecular Biotechnology and Genetics University of Lodz Lodz Poland; ^4^ Department of Tumor Biology Medical University of Lodz Poland

**Keywords:** airway remodelling, asthma, curcumin, inflammation, natural compounds, rhinovirus

## Abstract

Bronchial epithelial cells and fibroblasts play an essential role in airway remodelling, due to their protective and secretory functions. There are many studies proving that infection caused by human rhinovirus may contribute to the process of airway remodelling. The beneficial properties of curcumin, the basic ingredient of turmeric, have been proved in many studies. Therefore, the aim of this study was the evaluation of curcumin immunomodulatory properties in development of airway remodelling. Fibroblasts (WI‐38 and HFL1) and epithelial cells (NHBE) were incubated with curcumin. Additionally, remodelling conditions were induced with rhinovirus (HRV). Airway remodelling genes were determined by qPCR and immunoblotting. Moreover, NF‐κB, c‐Myc and STAT3 were silenced to analyse the pathways involved in airway remodelling. Curcumin reduced the expression of the genes analysed, especially MMP‐9, TGF‐β and collagen I. Moreover, curcumin inhibited the HRV‐induced expression of MMP‐9, TGF‐β, collagen I and LTC4S (*p *< 0.05). NF‐κB, c‐Myc and STAT3 changed their course of expression. Concluding, our study shows that curcumin significantly downregulated gene expression related to the remodelling process, which is dependent on NF‐κB and, partially, on c‐Myc and STAT3. The results suggest that the remodelling process may be limited and possibly prevented, however this issue requires further research.

## 1 INTRODUCTION

Airway remodelling changes include a thickened reticular basement membrane, dysregulated extracellular matrix (ECM) protein deposition and increased vasculature. Clinically, these features significantly decrease the quality of life of asthmatic patients. The mechanisms regulating airway remodelling changes and the order in which these changes develop remain poorly understood.^1^, ^2^ Traditionally, remodelling was thought to occur as a result of many years of chronic airway inflammation, but there is now clear evidence that airway remodelling begins in early childhood and can be present even before the clinical diagnosis of asthma has been established.^3^

Human rhinovirus (HRV) infections promote the expression of factors that have been associated with airway damage and remodelling. Repeated HRV‐induced respiratory illnesses during infancy and early childhood are strongly associated with an increased risk of subsequent asthma.^4^ Recurrent human rhinovirus infections are a potential stimulus for remodelling, which leads to the hypothesis that HRV infections may play a central role in the start of the airway remodelling leading to asthma. Recent studies have identified a number of potential pathways that could causally link recurrent HRV infection of the lower airway with airway remodelling and asthma development. Recent studies have identified a number of potential pathways that could causally link recurrent HRV infection of the lower airway with airway remodelling and asthma development. Leigh et al.^5^ presented a hypothesis that early childhood HRV infections might play a role in the development of the airway remodelling which manifests even before the confirmed diagnosis of asthma. Rhinovirus has been detected in subepithelial layers and cells, including fibroblasts in asthmatic airways, probably because of a disrupted and inflamed epithelium.^6^ It was recently found that fibroblasts from asthmatic patients enhance the replication of rhinovirus and induce a subsequent vigorous proinflammatory response with IL‐6 and IL‐8 production.^7^ There is also a confirmation that HRV infection of the airway epithelium both *in vitro* and *in vivo* results in robust upregulation of IL‐11 production.^8^ This suggests that HRV infection of airway epithelial cells is capable of upregulating a number of mediators implicated in the airway remodelling process.

Furthermore, such rhinovirus replication was augmented in TGF‐β–treated fibroblasts from asthmatic patients.^9^ The cytopathic effects of viral infection on epithelial cells predispose to an acute inflammatory response and could enhance airway remodelling.^10^ Rhinovirus infection also induces cell cytotoxicity and thereby reduces the cell proliferation rate resulting in an impaired repair process of the bronchial epithelial cells.^11^ Skevaki et al.^12^ demonstrated that RV‐specific basal FGF release is time‐ and dose‐dependent and results from both transcriptional upregulation and release from epithelial cells upon induction of cytotoxicity.

Curcumin is a polyphenolic compound derived from roots of popular Indian turmeric plant and used as a spice, but also in food colouring. The beneficial aspects of curcumin intake have been shown previously in many studies,^13^, ^14^, ^15^ and curcumin is very popular food ingredient, recommended by WHO at a dosage up to 3 mg per kg of body weight per day.^16^ However, its poor solubility and rapid biotransformation to inactive metabolites limit the utility of formulated curcumin; therefore, products that provide >100‐fold better absorption than unformulated curcumin are considered highly bioavailable.^17^ Immunomodulatory properties of curcumin have been reported recently,^18^ and benefits of curcumin in chronic phase of asthma have been investigated due to the complexity and involvement of many factors in the disease. Curcumin, having many beneficial properties, can be used as a potential therapeutic drug for the treatment of asthma. In Chauhan's study,^19^ intranasal curcumin effectively attenuated structural changes in asthmatic mice airways which occur either as a result of chronic allergic airway inflammations, injury or the repair process which leads to airway remodelling. It effectively suppressed recruitment of inflammatory cells to the lungs compared to dexamethasone. Moreover, the study also proved the effect of intranasal curcumin on extracellular matrix depositions and substantial reduction in the replacement of epithelial cells by goblet cells.

Curcumin also affects the level of metalloproteinases (MMPs) in extracellular sites by modulating the inducers, such as growth factors and cytokines (IL‐1α, IL‐1β, TNF‐α, TNF‐β, HGF etc.), occurring primarily at the transcriptional level and initiated by the binding of the stimulating factor to its cell surface receptor.^20^

There are only a few studies concerning the action of curcumin in asthma, but based on the literature, it might be concluded that curcumin is worth further studies in this disease as an anti‐inflammatory agent.

The aim of the study was to evaluate the curcumin effect on the airway remodelling‐related gene expression in the context of rhinovirus infections, HRV‐2 and HRV‐16, as the factor which induces changes leading to airway remodelling. We also analysed the action of curcumin under conditions of the knockout of transcription factors—NF‐κB, c‐myc and STAT3.

## 2 MATERIALS AND METHODS

### 2.1 Cell cultures

Fibroblast cell lines—WI‐38 and HFL1—were purchased from Sigma‐Aldrich (St. Louis, MO USA) and grown in EMEM medium (WI‐38) and HAM's12 medium (HFL1) with 10% foetal bovine serum, 2 mM of L‐glutamine, 1% of non‐essential amino acids and standard Penicillin‐Streptomycin solution (Sigma‐Aldrich, St. Louis, MO). The epithelial cell line—NHBE—was purchased from Lonza (Lonza Walkersville Inc. MD, USA) and cultured in BEGM Bronchial Epithelial Cell Growth Medium BulletKit (Lonza Walkersville Inc. Walkersville, MD, USA). All the experiments (*n* = 6) were performed after reaching 80–90% confluence (passage three to nine) by the cells. The viability of the cells was assessed by adding 10 µl of Presto Blue (BD Pharmingen, Franklin Lakes, NJ, USA), and the absorbance was measured at 570 nm.

### 2.2 Virus preparation and cell infection

Human rhinovirus (HRV) 16 and HRV‐2 were purchased from the European Collection of Authenticated Cell Cultures (*ECACC*, Salisbury, UK). Ohio HeLa cells were infected until cytopathic effects were observed (multiplicity of infection (MOI) of 1, established on the base of literature^21^, ^22^, ^23^). HRV specimens were exposed to the temperature of 58°C for one hour in order to inactivate the virus particles, which was subsequently confirmed by a lack of HRV replication.

The target fibroblast and epithelial cells were infected by the addition of 50 μL vehicle (medium) or HRV16. The cells were incubated for 24 hours (33°C, 5% CO_2_).^24^

### 2.3 Experimental procedure

The cultures were exposed to the HRV‐2 (minor serotype) and HRV‐16 (major serotype) virus for 24 hours (33°C, 5% CO_2_). Following this, or before infection, the cells were incubated with curcumin (2 μM) for 24 hours (37°C, 5% CO_2_). The controls were treated with medium only. We have chosen one HRV serotype from each group in order to evaluate potential differences between them.

### 2.4 RNA isolation and cDNA synthesis

Total RNA was isolated from the cells by using a Total RNA mini kit (A&A Biotechnology, Gdynia, Poland). The RNA was subsequently purified and stored at −80°C. Reverse transcription using 1 μg of total RNA was performed using a High Capacity cDNA kit (Applied Biosystems, Foster City, CA, USA). The procedures were performed according to the producer's protocols.

### 2.5 Gene expression analysis

The changes in the expression of metalloproteinase‐9, transforming growth factor β1 (TGF‐β1), collagen I, disintegrin and metalloproteinase domain‐containing protein 33 (ADAM33), chitinase‐3‐like protein 1 (YKL‐40), relaxin/insulin‐like family peptide receptor 1 (RXFP1), leukotriene C4 synthase (LTC4S) and alpha‐SM‐actin (α‐SMA) were assessed utilizing qPCR. TaqMan gene expression assays were used for the selected genes: MMP‐9—Hs00957562_m1, TGF‐β1—Hs00998133_m1, collagen I—Hs00164004_m1, ADAM33—Hs00905552_m1, YKL‐40—Hs01072228_m1, RXFP1—Hs01073145_m1, LTC4S—Hs01073145_m1, α‐SMA—Hs05005339_m1, and β‐actin—Hs99999903_m1 (Life Technologies, Carlsbad, CA). Each sample was measured in triplicate using the TaqMan analyzer, and the 2^−ΔΔCt^ method was used to calculate gene expression. The results were normalized to an endogenous reference gene (β‐actin—Hs99999903_m1). LTC4 synthase was evaluated as an inflammation marker and α‐SMA—as fibroblast‐myofibroblasts transformation marker. By comparing RQ (relative quantification, 2^−ΔΔCt^), the fold change in mRNA expression was calculated.

### 2.6 Protein isolation and immunoblotting

RIPA protein extraction buffer (Sigma‐Aldrich, St. Louis, MO, USA), supplemented with a protease inhibitor cocktail (Sigma‐Aldrich, St. Louis, MO, USA), was used to extract total protein, determined subsequently by the BCA Protein Assay Kit (Pierce Thermo Scientific, USA). The electrophoresis was performed utilizing 10 µg of protein in denaturing polyacrylamide 4–20% NuPage gel (Invitrogen, Carlsbad, CA, USA) for 60 minutes (140 V and 110 mA). After that, the specimens were transferred into a nitrocellulose membrane with the eBlot Protein Transfer System (Genscript, Piscataway, NJ, USA).

The membrane was incubated for one hour at room temperature with 5% nonfat milk dissolved in TBST. Subsequently, the membrane was incubated with primary antibodies (mouse) for 12 hours at 4°C and then with secondary anti‐mouse IgG secondary antibodies (goat), conjugated with alkaline phosphatase for 90 minutes at room temperature. All the antibodies were purchased from Santa Cruz Biotechnology, Dallas, USA. The bands on the membrane were developed using a BCIP/NBT alkaline phosphatase substrate (Merck Millipore, Darmstadt, Germany) and after that analysed with Image J 1.49 software (Wayne Rasband, National Institutes of Health, Bethesda, MD).

### 2.7 siRNA silencing of transcription factors

For the knockdown of selected genes (NF‐κB, c‐Myc and STAT3), a Silencer siRNA Transfection Kit was used, according to the manufacturer's instructions (Thermo Fisher Scientific, Waltham, MA USA). The cells were treated with 20 nM siRNA mixture against NF‐κB (NCBI accession no. NM_001145138.1), c‐Myc (NCBI accession no. NM_002467.4) and STAT3 mRNA (NCBI accession no. NM_003150.3) (Thermo Fisher Scientific, Waltham, MA USA) for 48 hours. The same concentration of scrambled siRNAs was used as the negative control. The knockdown efficiency was evaluated after 48 hours of transfection. The measure of gene knockdown was analysed according to the manufacturer's protocol by qPCR.

### 2.8 Statistical analyses

The obtained results were analysed with software Statistica software (StatSoft, Tulsa, OK). The Shapiro‐Wilk test and Levene's test were, respectively, utilized to check the distribution of data as well as the equality of variances. Significant changes were calculated using the ANOVA test with the appropriate post hoc tests as a multiple comparison procedure. *p* values <0.05 were considered to be statistically significant.

## 3 RESULTS

### 3.1 The effect of curcumin on airway remodelling gene expression

**FIGURE 1:**
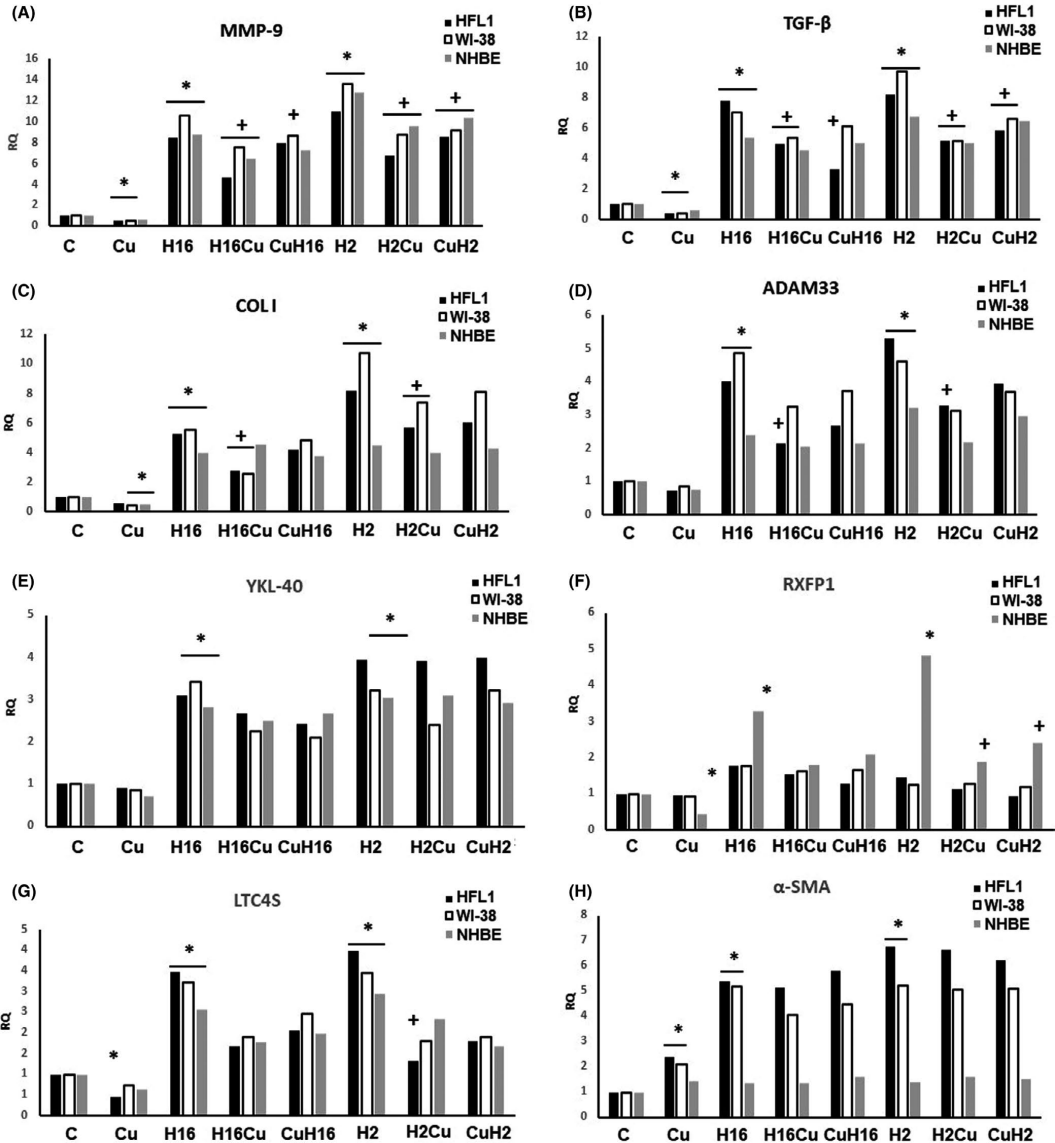
Effect of curcumin on rhinovirus‐triggered mRNA expression of airway remodelling‐involved genes. Rhinovirus 2 (minor serotype) and 16 (major serotype) were used. Curcumin (Cu) was added before or after rhinovirus‐16 infection (CuH16 or H16Cu respectively). Curcumin decreased the expression of MMP‐9 (A), TGF‐β (B), collagen I (C), LTC4S (G) and α‐SMA (H) in fibroblasts (*p *< 0.05) as well as the expression of collagen I (C) and RXFP1 (F) in epithelial cells (*p *< 0.05). Rhinoviruses (H16 for rhinovirus 16 and H2 for rhinovirus 2) increased the expression of all genes analysed in fibroblasts, except for RXFP1 (F), in which the effect was only in epithelial cells (*p* < 0.05). Curcumin abolished the effect of rhinoviruses by decreasing the mRNA expression of MMP‐9 (A), TGF‐β (B), collagen I (C), ADAM33 (D) and LTC4S (G) in fibroblasts, whereas in the case of epithelial cells, its action was observed in the RXFP1 expression only (F, *p *< 0.05). Additionally, curcumin added to already‐infected cells presented its effect contrary to conditions under which curcumin was pre‐incubated prior to infection. *p < 0.05 in comparison with the control sample, +*p *< 0.05 in comparison with the rhinovirus sample (HRV‐2 or HRV‐16 respectively); C—control sample with medium only

**FIGURE 2:**
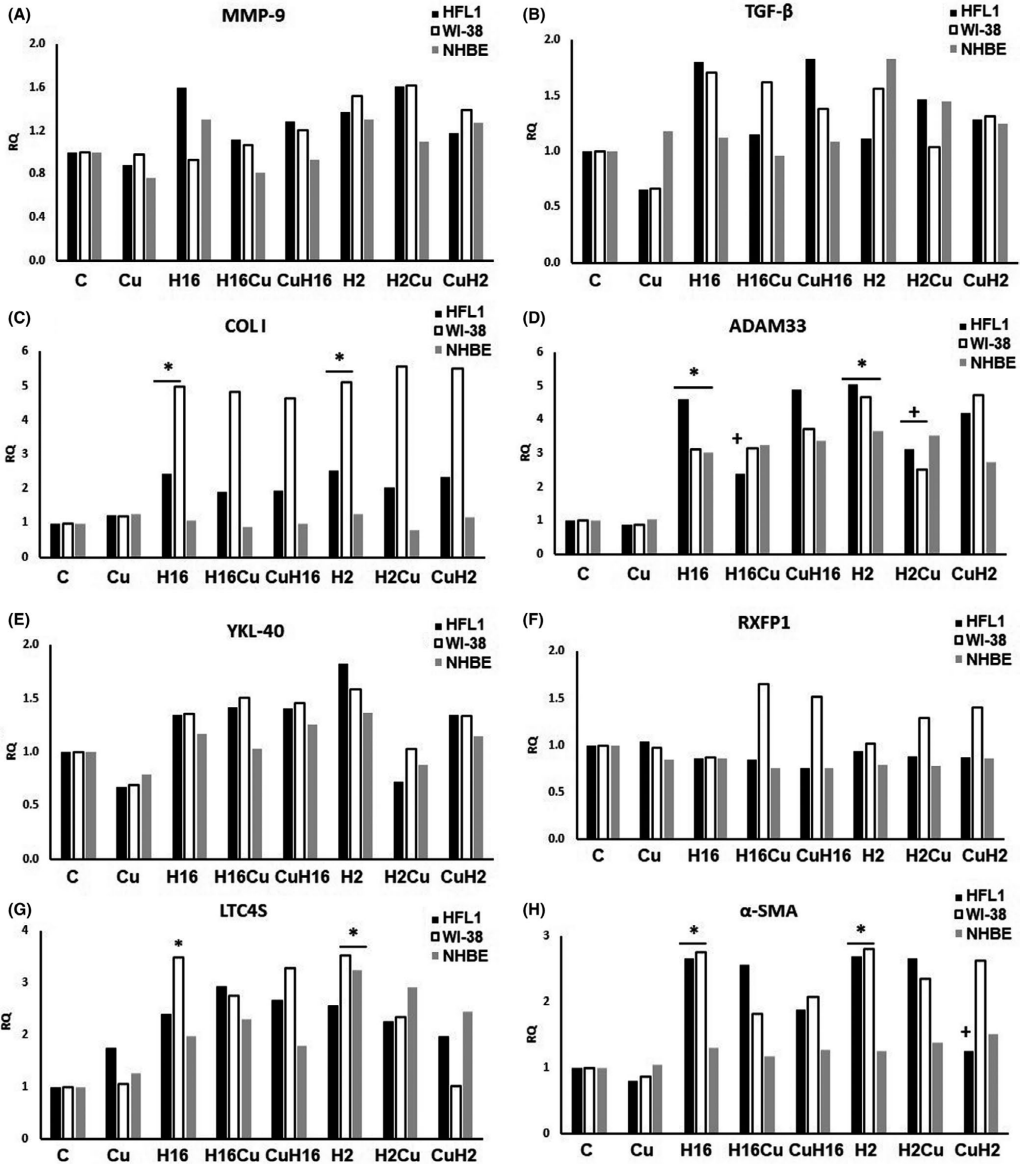
NF‐κB silencing effect on curcumin activity under rhinovirus infection conditions. Rhinovirus 2 (H2 in the Figure, minor serotype) and 16 (H16 in the Figure, major serotype) were used. Curcumin (Cu) was added before or after rhinovirus‐16 infection (CuH16 or H16Cu respectively). NF‐κB silencing eliminated effects of HRV and curcumin in MMP‐9 (A), TGF‐β (B), YKL‐40 (E) and RXFP1 (F), (*p *> 0.05). The knockout did not change the ADAM33 expression in comparison with cells expressing NF‐κB (D, *p *< 0.05). *p < 0.05 in comparison with the control sample, +*p *< 0.05 in comparison with the rhinovirus sample (HRV‐2 or HRV‐16 respectively); C—control sample with medium only

Firstly, we aimed at the evaluation of the curcumin effect on different types of cells, fibroblasts (HFL1, WI‐38) and epithelial cells (NHBE). Curcumin significantly deceased the expression of MMP‐9, TGF‐β and collagen I in fibroblasts (*p *< 0.05, Figure 1A–C, Figure 2A–C) and increased the expression of α‐SMA in these cells (*p *< 0.05, Figure 1H). These results are surprising, as α‐SMA, a marker of airway remodelling in asthma, and MMP‐9, TGF‐β and collagen I play a role in airway remodelling. Moreover, decrease in MMP‐9 might be a marker of soothing of inflammation process. TGF‐β is strongly implicated in airway remodelling and is released by eosinophils at the site of allergic inflammation and it also promotes metalloproteinase‐9 production, so these genes expression is connected. Curcumin decreased the RXFP1 expression in epithelial cells, with no effect on the expression of other genes analysed (Figure 1F, *p* < 0.05, Figure 2F). One of the major sites for RXFP1 expression is bronchial epithelium, and epithelial cells are highly secreting. They can have a big impact on the underlying mesenchymal cells, including fibroblasts.

### 3.2 The effect of curcumin on the modulation of gene expression induced by two rhinovirus serotypes

Both serotypes of rhinoviruses, HRV‐16 and HRV‐2, significantly increased the expression of *MMP*‐*9*, *TGF*‐*β*, collagen I, *ADAM33*, *YKL*‐*40* and *LTC4S* in fibroblasts and in epithelial cells (*p *< 0.05, Figure 1A–E and G, Figure 2A–E and G). Such results confirm the role of HRV as a triggering factor—changes related to airway remodelling, as all of these genes are documented to be increased in this process. Moreover, *ADAM33*, *TGF*‐*β1* and *YKL*‐*40* are the candidate genes for asthma in the context of airway remodelling. ADAM33 plays a critical role in cell adhesion, proliferation, differentiation, signalling, apoptosis and inflammatory responses, and in limited studies of airway remodelling, serum YKL‐40 levels have correlated with the thickness of subepithelial basement membrane in bronchial biopsy specimens.^25^

Additionally, the effect of rhinovirus was observed in the expression of *α*‐*SMA* only in fibroblast cells (*p *< 0.05, Figure 1H) and in the expression of *RXFP1* only in epithelial cells (*p *< 0.05, Figure 1F). Interestingly, the effects of HRV‐2 were generally stronger in each cell type (Figure 1A–H and Figure 2A–H). This serotype belongs to a minor HRV group, and these results show the difference between effects of the serotypes of both viruses.

### 3.3 Analysis of the differences in the order of curcumin stimulation in rhinovirus infections

Half of the samples were HRV‐induced cells and then stimulated with curcumin, and the other half of the samples were infected with HRV cells after pre‐incubation with curcumin. It appeared that curcumin added to HRV‐infected cells (no difference whether it was HRV‐2 or HRV‐16) has exerted a stronger effect than added at first, before HRV infection.

Curcumin significantly decreased the HRV‐induced *MMP*‐*9* expression in fibroblasts and epithelial cells, in comparison with HRV samples (Figure 1A, *p* < 0.05, and Figure 2A); however, in the case of HRV infection, the effect of curcumin pre‐incubations was also statistically significant (*p *< 0.05, Figure 1A). A similar effect was observed in the *TGF*‐*β* expression; however, curcumin was effective in decreasing the HRV‐2 and HRV‐16 effect only in fibroblasts (*p *< 0.05, Figure 1B). For collagen I, *ADAM33* and *LTC4S*, their expressions were decreased in comparison with HRV only, when curcumin was added to HRV‐induced fibroblasts, but most importantly, in case of collagen, the result was confirmed on the protein level (Figure 1C, D, G, *p* < 0.05 and Figure 2C, D and G). Oppositely, the same effect of curcumin but in epithelial cells was observed only in the RXFP1 expression (HRV‐2) (Figure 1F, *p* < 0.05, and Figure 2F). These results suggest that curcumin decrease HRV‐induced, remodelling‐involved genes expression.

### 3.4 NF‐κB, silencing influences transcription of the genes contributing to airway remodelling process

In order to establish the role of chosen transcriptional factors in the remodelling process, we silenced three of them and analysed the curcumin effect in the context of HRV‐2 and HRV‐16 infections. NF‐κB is important in signalling pathways, also important in airway remodelling in asthma. It is activated *via* several signalling pathways, leading to a signalling cascade that amplifies inflammation.^26^

**FIGURE 3:**
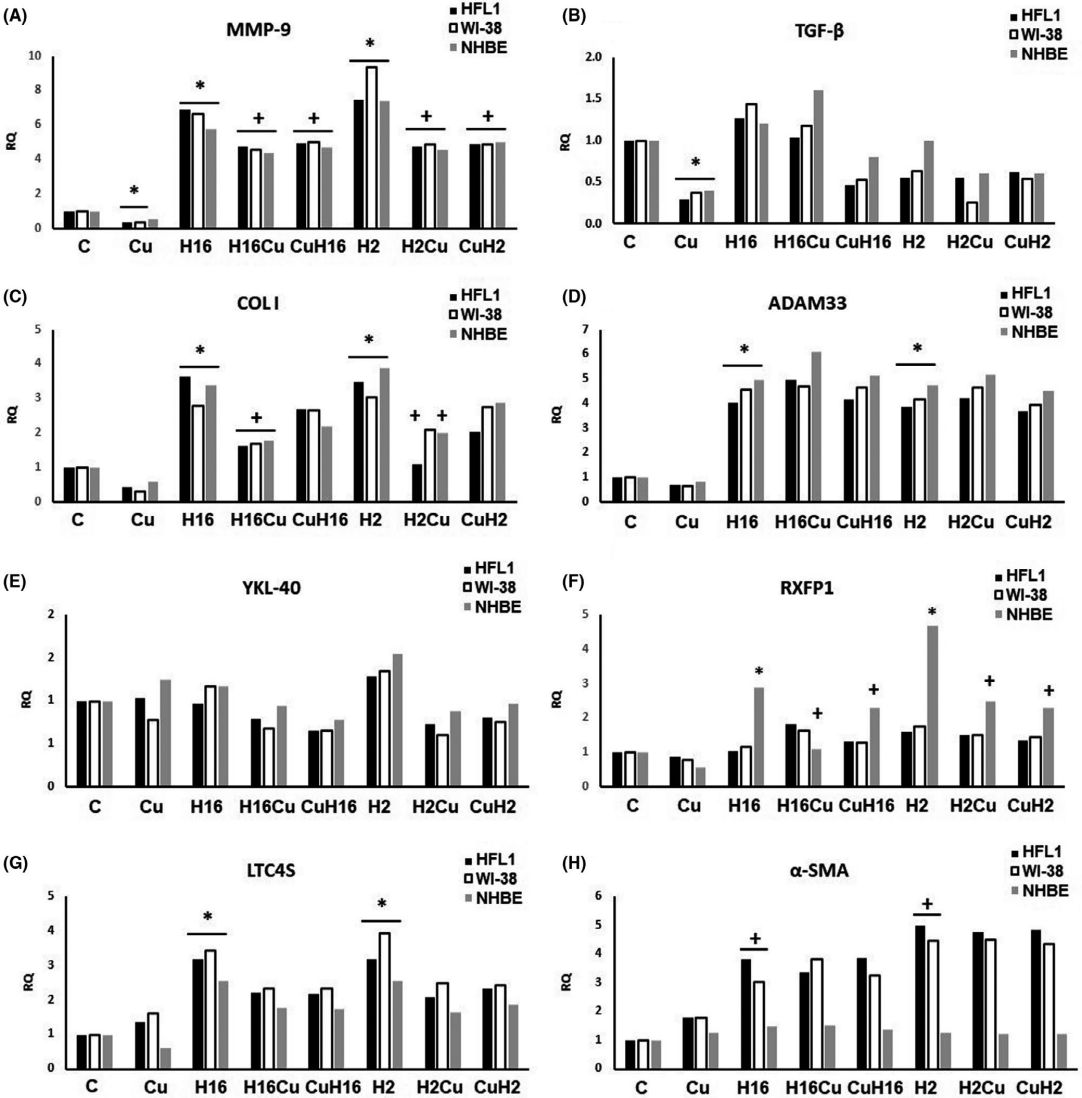
Action of curcumin (Cu) in c‐Myc knockout cells under Rhinovirus infection conditions. Rhinovirus 2 (H2 in the Figure, minor serotype) and 16 (H16 in the Figure, major serotype) were used. The knockout of transcription factor c‐Myc, abolished rhinovirus 2 and 16 stimulating effect on TGF‐β (B), and YKL‐40 (E). Curcumin ability to decrease the HRV‐induced mRNA expression was also inhibited in the ADAM33 (D) and LTC4 (G) expression (*p *> 0.05). Interestingly, under condition of c‐Myc silencing, curcumin appeared to be more effective in decreasing the HRV‐induced RXFP1 expression (F, *p *< 0.05). *p < 0.05 in comparison with the control sample, +*p *< 0.05 in comparison with the Rhinovirus sample (HRV‐2 or HRV‐16 respectively). CuH2 and CuH16—curcumin added before rhinovirus infections, CuH2, CuH16—curcumin added 24h after infection, C—control sample with medium only

*NF*‐*κB* knockout eliminated all the changes in the expression of *MMP*‐*9*, *TGF*‐*β*, *YKL*‐*40* and *RXFP1* in each of the cell types (*p *< 0.05, Figure 3A, B, E and F). However, both serotypes of HRV analysed, significantly increased collagen I and α‐SMA expression in fibroblasts (*p *< 0.05, Figure 3C and H), as well as *ADAM33* and *LTC4S* in fibroblasts and epithelial cells (*p *< 0.05, Figure 3D and G). Interestingly, pre‐incubation with curcumin effectively inhibited the effects of HRV‐2 in the expression of *α*‐*SMA* and the effects of HRV‐16 in the case of *ADAM33* expression, which were the only effects of curcumin in this set of experiments (*p *< 0.05, Figure 3H and D respectively).

### 3.5 c‐Myc knockout affects influences transcription of the genes contributing to airway remodelling process

**FIGURE 4:**
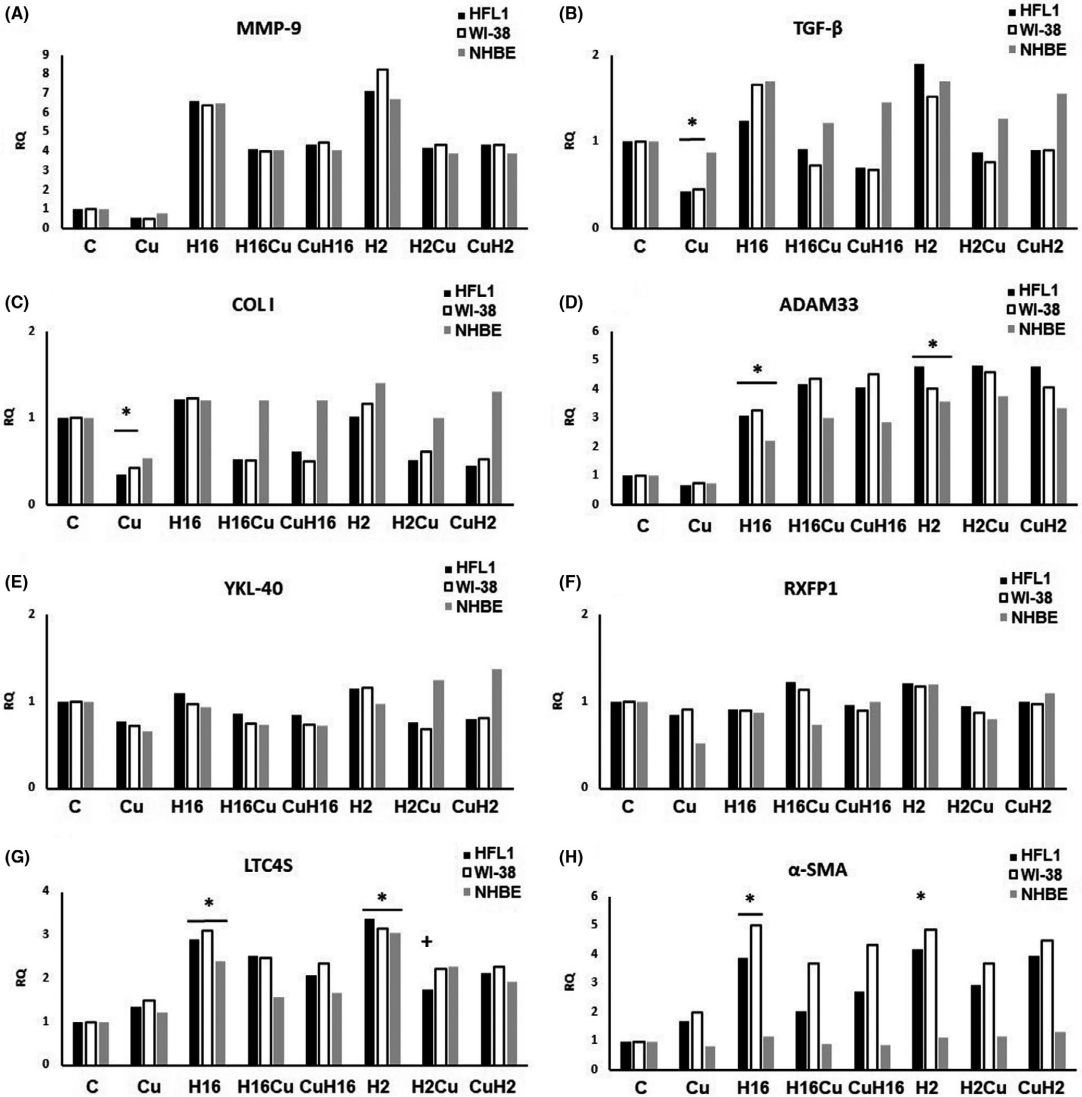
Effects of STAT3 knockout on curcumin (Cu) abilities under Rhinovirus infection conditions. Rhinovirus 2 (H2 in the Figure, minor serotype) and 16 (H16 in the Figure, major serotype) were used. In comparison with the conditions without knockout, the TGF‐β (B) and collagen I (C) expression was decreased by curcumin (Cu) only, without any effect of HRV‐2 or HRV‐16. Also RXFP1 expression changes were not observed, either after rhinoviruses, or after curcumin in bot cell types. In the case of ADAM33 (C), LTC4 (G) and α‐SMA (H), curcumin did not appear to cause any effect (*p *> 0.05). *p < 0.05 in comparison with the control sample, +*p* < 0.05 in comparison with the rhinovirus sample (HRV‐2 or HRV‐16 respectively). CuH2 and CuH16—curcumin added before rhinovirus infections, CuH2, CuH16—curcumin added 24h after infection, C—control sample with medium only

**FIGURE 5:**
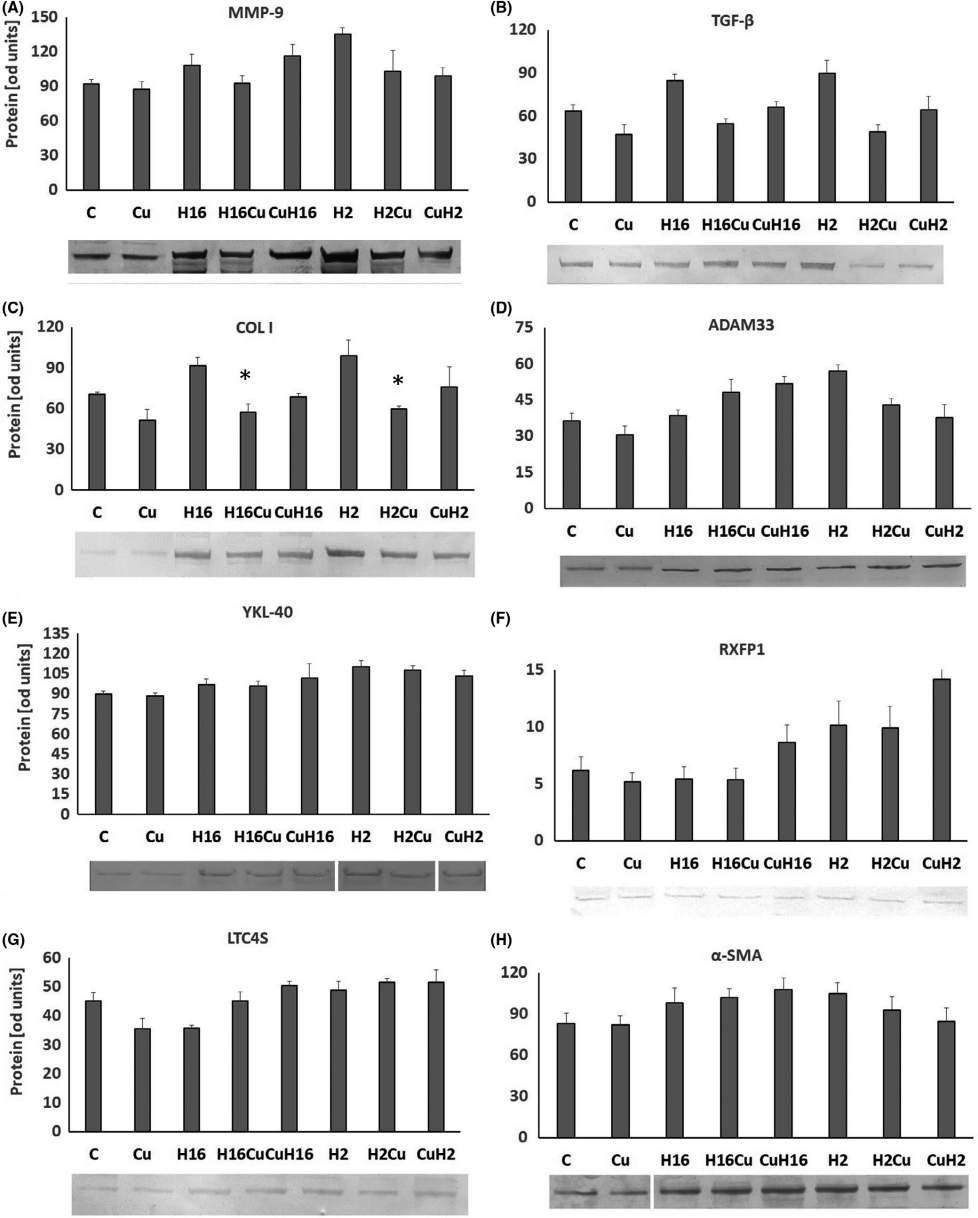
Immunoblot confirmation of curcumin effects. Representative Western blots of genes in which the statistical significance has been observed at the mRNA level. MMP‐9, TGF‐β, collagen I, YKL‐40 and α‐SMA are the results from HFL‐1 fibroblast cell line, ADAM33 and LTC4S—from WI‐38 cell line and RXFP1—from NHBE epithelial cell line, according to mRNA results obtained. Cu—curcumin, H16—rhinovirus 16, H2—rhinovirus 2. The Figures E and H are composite of separate bands in a lane. CuH and HCu mean the order of curcumin stimulation—before or after rhinovirus infection (with the number of serotype respectively)

Recent study has shown that upregulation of transcription factor c‐Myc may affect the asthma airway remodelling.^27^ c‐Myc expression is also upregulated in peripheral blood group 2 innate lymphoid cells (ILC2) of asthma patients. siRNA silencing of c‐Myc transcription factor gene appeared to affect the levels of expression of MMP‐9, collagen I, ADAM33, RXFP1 and α‐SMA upregulated by rhinoviruses: although the RQ levels were significant in comparison with the control sample, they were lower without knockout (Figure 4A, C, D, F, *p* < 0.05, and H, Figure 5A, C, D, E and G). We did not detect the changed TGF‐β expression (Figure 4B, Figure 5B). What is important, curcumin significantly decreased the MMP‐9 expression in fibroblasts and epithelial cells, causing also a decrease in the HRV‐induced expression in this gene with no difference in the order of stimulation (*p *< 0.05, Figure 4A). Similarly, curcumin decreased the HRV‐induced collagen expression in fibroblasts and epithelial cells (*p *< 0.05), with no effect, however, when added before HRV infection (*p *> 0.05, Figure 4C). We observed an upregulatory effect of HRV‐2 an HRV‐16 on the ADAM33, RXFP1 and LTC4S expression in fibroblasts and epithelial cells (Figure 4D, F and G, *p* < 0.05, Figure 5D, E and F), whereas the α‐SMA expression was increased only in fibroblasts (*p *< 0.05, Figure 4H). Similarly to the experiments without siRNA silencing, curcumin decreased the HRV‐induced expression of RXFP1in epithelial cells, with no difference in the order of stimulation (*p *< 0.05, Figure 4F). No changes were observed in YKL‐40 mRNA expression (Figure 4E).

### 3.6 The effects of STAT3 on airway remodelling‐related genes expression

**FIGURE 6:**
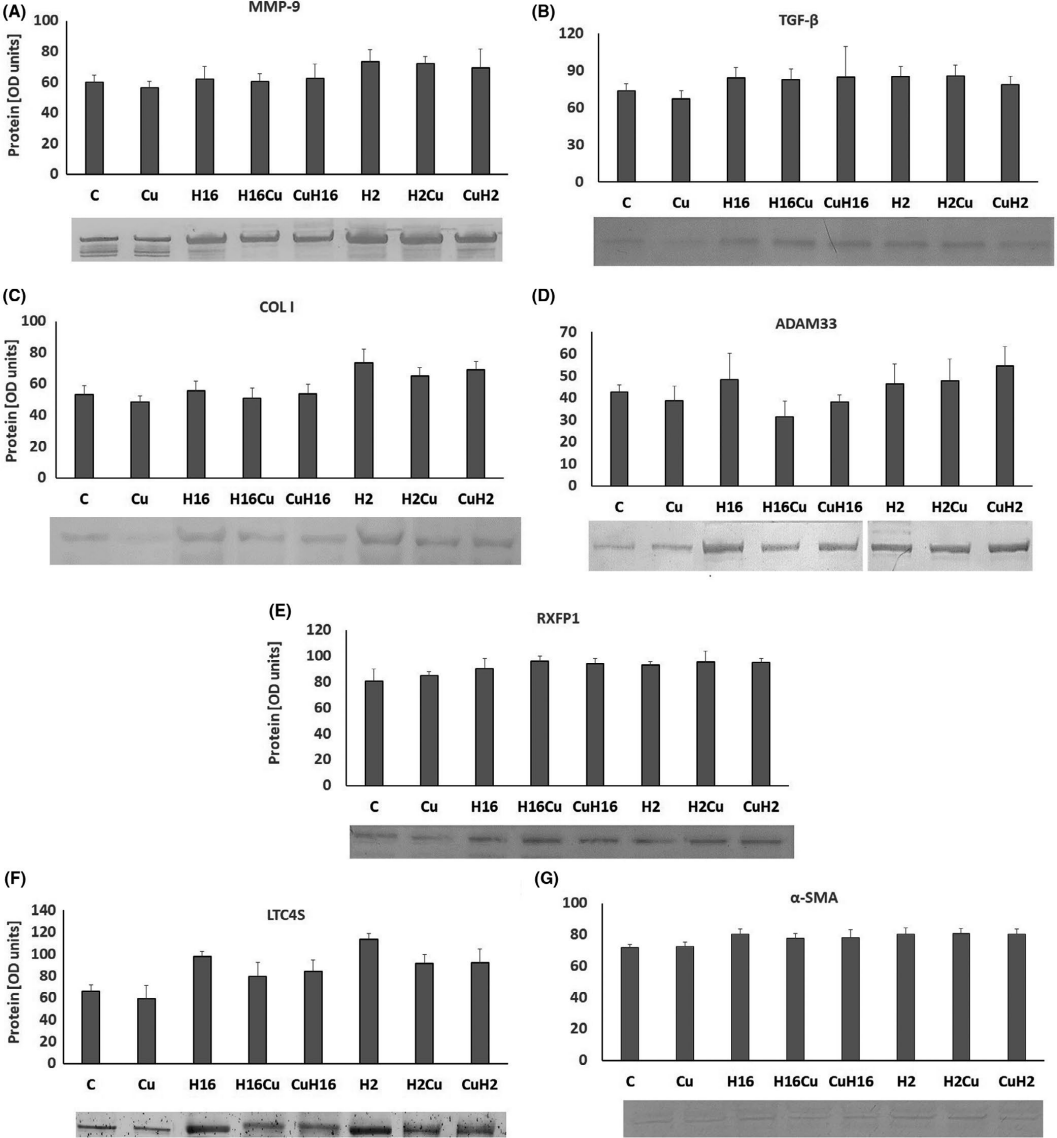
Box plot for protein expression levels from c‐Myc silenced cells. Representative Western blots of genes in which the statistical significance has been observed at the mRNA level. The blots are the results for HFL1, except for RXFP1, which was expressed in NHBE epithelial cells. The Figure D is a composite of separate bands in a lane. Cu—curcumin, H16—rhinovirus 16, H2—rhinovirus 2. CuH2 and CuH16—curcumin added before rhinovirus infections, CuH2, CuH16—curcumin added 24h after infection, C—control sample with medium only

**FIGURE 7:**
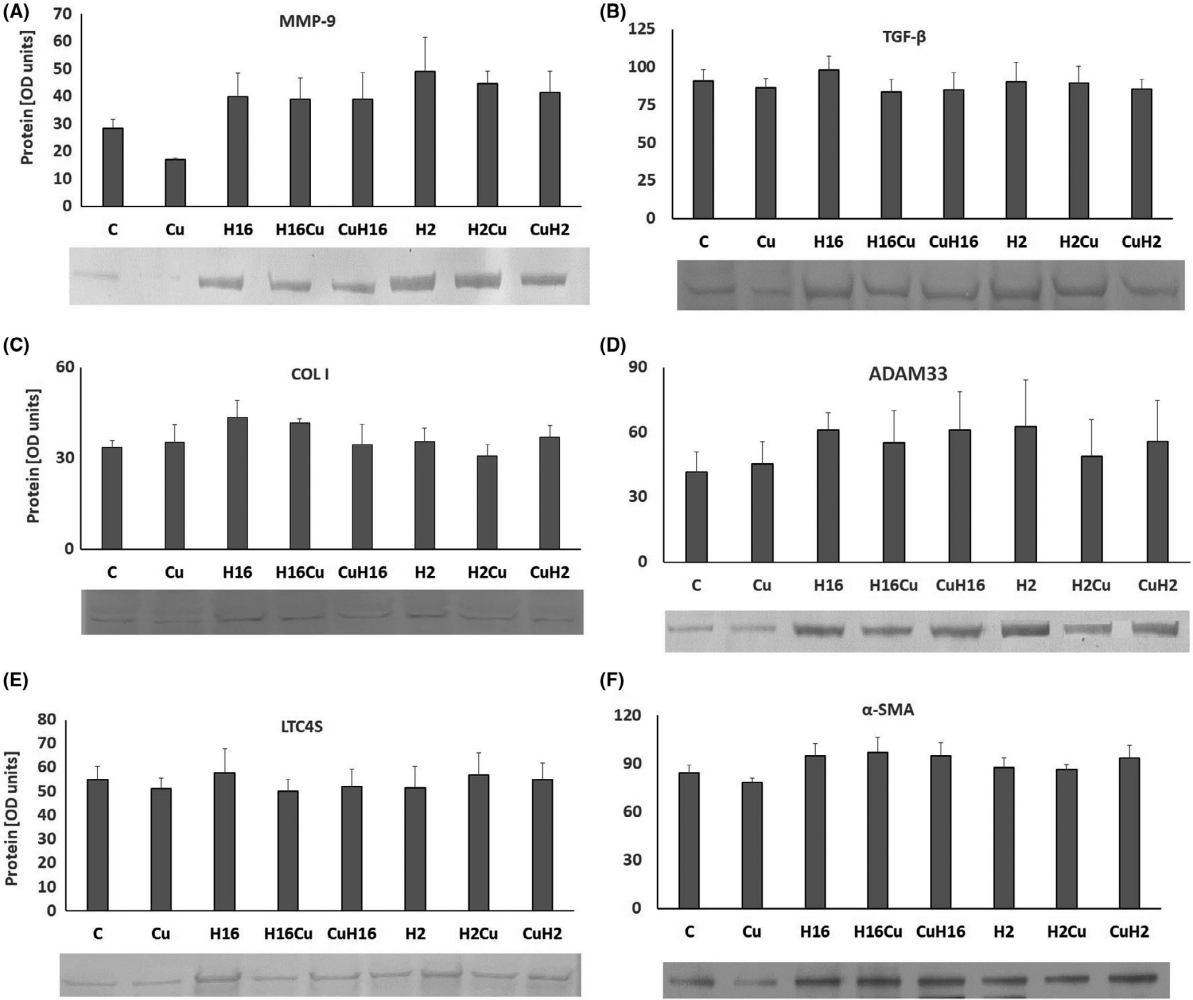
Protein expression measured by immunoblotting. Representative Western blots of genes in which the statistical significance has been observed at the mRNA level. The results are from HFL1 fibroblast cell line, according to mRNA results obtained. Cu—curcumin, H16—rhinovirus 16, H2—rhinovirus 2. H2Cu—curcumin added to rhinovirus‐2 infected cells, CuH2—curcumin added before the infection

*STAT3* knockout resulted in an increased expression of *MMP*‐*9* induced by HRV‐2 and HRV‐16, and this effect was inhibited by curcumin in fibroblasts and epithelial cells, with no difference in the order of stimulation (Figure 6A, *p* < 0.05, and Figure 7A). STAT3 transcription factor is critical for cytokine signalling and the acute phase response, but its role in asthma and airway remodelling is largely undefined. Upregulation of STAT3 was found in peripheral blood mononuclear cells and airway smooth muscle tissues from asthmatics and correlated with the increased cytokines levels. Inhibition of STAT3 signalling can remit the airway inflammation.^28^

*TGF*‐*β* and collagen I expressions in fibroblast cells showed significant decreases caused by curcumin only, with no result on rhinoviruses (Figure 6B, C and Figure 7B, C). On the other hand, rhinoviruses upregulated expressions of *ADAM33*, *LTC4S* (in fibroblasts and epithelial cells, Figure 6D, G and Figure 7D, E) and α‐SMA (in fibroblasts, Figures 6H, 7F), which were inhibited by curcumin only in the case of ADAM33 and α‐SMA (*p *< 0.05). We did not observe any effects in *YKL*‐*40* and *RXFP1* (*p *> 0.05, Figure 6E, F).

## 4 DISCUSSION

In the world literature, there is no clearly established knowledge about the regulation and potential pharmacological modulation of airway remodelling. Moreover, the lack of available literature reporting potential drugs that could slow down the process of airway remodelling makes it an issue of peculiar importance. In this study, we evaluated the modulation of airway remodelling‐related genes by curcumin in the presence of rhinovirus as an airway remodelling triggering factor.^29^ Curcumin was nontoxic for WI‐38, HFL1 and NHBE cells (IC_50_ > 80 μM, data not shown), consistent with previous reports.^30^, ^31^, ^32^

Curcumin appeared to be an effective modulator of the genes directly involved in airway remodelling and a significant decrease in the expression of TGF‐β, MMP‐9, collagen I and LTC4S. These results confirm its anti‐inflammatory effect, observed, however, only in fibroblasts (Figure 1A‐C and G). Our data are consistent with the literature; Chauhan et al. suggested that intranasal curcumin alleviated airway inflammation in mice,^19^ and Kumari et al.^33^ published data showing that curcumin inhibits the development of LPS‐induced remodelling through the modulation of MMP‐9 expression. Reduced TGF‐β by curcumin has been proven before,^34^, ^35^, ^36^ and curcumin pretreatment effectively blocking collagen I expression has been presented by Gaedeke et al..^37^

Although a large body of literature shows data confirming a positive role of curcumin against the development and maintenance of the airway remodelling process, there are limited data presenting curcumin action in the context of rhinoviruses. The recent study of XuChen et al.^38^ emphasized the need of investigating rhinovirus‐induced airway remodelling growth factors. In our research, we analysed the effects of curcumin as a pretreatment for viral infection, but also its action when applied to an existing viral infection. Interestingly, the order of application determined the effect, and in most cases, not the preventive action turned out to be most effective. Curcumin decreased rhinovirus‐induced genes related directly to airway remodelling, MMP‐9, TGF‐β, collagen I, YKL‐40, α‐SMA and ADAM33, but also LTC4S mRNA expression in fibroblasts. We showed a trend to reduce HRV‐induced gene expression in epithelial cells, but only in fibroblasts, these changes were significant. This was surprising, as airway epithelial cells and fibroblasts are the main cell types involved in the airway remodelling. Epithelial surface regulates the repair process *via* the extracellular matrix protein secretion and interactions with fibroblasts, which in turn actively release cytokines stimulating epithelial cell functions.^39^, ^40^ Moreover, it must be kept in mind that the pathological changes in the airway are coordinated by crosstalk between different cell types, which may differ because of their sensitivity of the cells to various stimuli.^41^

In our study, RXFP1 was the only gene whose expression was significant in epithelial but not in fibroblast cell lines (Figure 1F). These results, however, seem to be justified by studies conducted by Royce et al. who indicate that one of the main sites of the RXFP1 expression is the bronchial epithelium. Thus, the fibroblast expression of RXFP1 appears to be only a secondary effect of epithelial influential capability on the underlying cells.^42^, ^43^ Additionally, we report here that the same effect of curcumin, observed only in epithelial cells, appears also in the absence of *c*‐*Myc*, but not when *NF*‐*κB* and *STAT3* were silenced (Figures 3F and 6F respectively). We observed no effect in both fibroblast cell lines utilized (Figures 1F, 3F, 4F and 6F). Tan et al. showed that the RXFP1 expression is decreased in pulmonary fibrosis; therefore, our results may suggest that these transcription factors are specifically involved in the airway remodelling process. Although we cannot answer this question definitively, the literature reports demonstrate that the downregulation of RXFP1 expression in fibrotic tissues may be the reason for inefficacy of relaxin‐based therapy,^44^, ^45^, ^46^ and this issue requires further research as RXFP1 seems to be an interesting therapeutic target.^45^

The important finding of our study is difference in the action of curcumin due to the context of its administration. For the first time, we show that the effects of curcumin depend on the conditions to which it was added—before or during the infection. Curcumin added to the infected cells showed a stronger inhibitory effect. This was seen in the expression of *TGF*‐*β*, *MMP*‐*9* and collagen I, the genes most involved in the remodelling process. One of the reasons of such results might be the ability to inhibit NF‐κB. Rhinovirus infections have been reported to enhance the *NF*‐*κB* expression.^22^ Though the results published concerned epithelial cells, rhinovirus infection triggered inflammatory pathways also in other types of cells, so possibly this scenario might be applied also to fibroblasts. Curcumin added to already‐infected cells may effectively inhibit NF‐κB, resulting in downregulation of the genes controlled by this transcription factor. To confirm this supposition, we knocked out NF‐κB and analysed curcumin action. As shown in Figure 3, the effect of curcumin alone or in the infectious condition was inhibited after *NF*‐*κB* knockout. This shows that curcumin acts through this transcription factor on *MMP*‐*9*, *TGF*‐*β* and *COLI* (Figure 3A–C respectively). Additionally, as a confirmation of this hypothesis, we observed lower levels of expression in HRV samples; HRV‐induced levels of collagen I, *ADAM33*, *α*‐*SMA* and *LTC4S* were also lower than in samples without *NF*‐*κB* knockout. In asthma, biological effects of curcumin are associated with several signalling pathways which lead to a decrease in the inflammatory cytokine production and cell infiltration as well as to the inhibition of airway hyperresponsiveness.^47^ NF‐κB activation within the epithelium has been implicated in the pathogenesis of asthma, yet the exact role of epithelial NF‐κB in allergen‐induced inflammation and airway remodelling remains unclear. Nevertheless, NF‐κB is known to contribute to changes occurring during the remodelling process. Results presented by Tully et al. describe a critical role for nonciliated airway epithelial NF‐κB in promoting inflammation and fibrotic remodelling following extended challenges of house dust mite.^48^

Interestingly, our results suggest that curcumin might also act *via* the c‐Myc transcription factor. In our study, the inhibition of *c*‐*MYC* with siRNA resulted in a decrease in RQ levels of MMP‐9 and collagen I (Figure 3A and C). Little is known about the possible contribution of c‐Myc to airway remodelling; however, Perry et al. found that TGF‐β increases the expression of *c*‐*MYC* in ASM cells from severe asthmatics, with no effect on ASM cells from non‐severe asthmatics. Moreover, **i**nhibition of c‐Myc attenuated TGF‐β‐induced cell proliferation in ASM cells of asthmatics,^49^ which is confirmed by our research. Simon et al. confirmed previously published results that Src and STAT3 are necessary for c‐*myc* induction in fibroblasts. The authors claimed that the induction of c‐*myc* gene expression by itself may contribute to the upregulation of cyclin D, which is probably involved in airway remodelling process.^50^ c‐Myc and STAT3 are interrelated transcription factors; it has been reported that c‐Myc is necessary for STAT3 transcription and a downstream effector of Stat3 signalling.^51^, ^52^ Moreover, in human lung cancer cells, c‐Myc is a STAT3‐regulated gene.^53^ The previously published study by Alexandrow et al. presented a hypothesis that one of the pathways by which curcumin acts on the bronchial epithelium is the inhibition of the STAT3 pathway.^54^ Having established a potential participation of c‐Myc, we therefore evaluated the effect STAT3 knockout on the curcumin effect in the context of HRV infections. The influence of STAT3 silencing resulted in the expression of collagen I, RXFP1 and YKL‐40. Interestingly, these conditions resulted in the curcumin inhibitory effect of α‐SMA expression, and TGF‐β has shown to be at basal levels. Normally, STAT3 acts positively on cell proliferation as well as on cell survival; nevertheless, it may contribute to various diseases and have pathological results when is permanently activated.^55^ Moreover, STAT3 activation has been shown in fibrotic tissues, but its role in their triggering and development is not clear. STAT3 is one of the alternative transcription factors that have been reported to bind to the Collagen Type I Alpha 2 chain gene promoter, in response to TGF‐β stimulation.^56^ Studies on intestinal and kidney cells confirm the role of STAT3 in fibroblast ECM remodelling and deposition in the course of fibrosis.^57^, ^58^ The study of Papaioannou et al. suggests that STAT3 is required in lungs for the increased COL1A2 expression observed in myofibroblasts, and furthermore, that it blocks TGF‐β signalling, TGF‐β‐induced myofibroblast differentiation and matrix remodelling.^59^ These results are in agreement with our study, as we demonstrated that STAT3 knockout significantly reduced the expression of TGF‐β as well as collagen I (Figure 6B‐C). Moreover, recent studies by Chakraborty et al. revealed that STAT3‐deficient fibroblasts are less sensitive to the pro‐fibrotic effects of TGFβ.^60^ Curcumin as a STAT3 inhibitor constitutes a therapeutic strategy which may significantly influence the modulation of the pathways leading to airway remodelling. The obvious limitations of our study are cell culture experimental model, and the fact that single viral infection might not be sufficient to induce full range of changes present in airway remodelling development. However, there is scarce literature describing directly this issue and this model seems to be suitable for initial research. Moreover, the aim of this study was to demonstrate the effect of curcumin on the expression of remodelling genes and to determine the effect of the order of its administration on the infection. The results obtained suggest that these studies should be extended and in order to establish more precisely the beneficial remodelling potential of curcumin. Curcumin as a feasible drug could be taken orally, for example in the form of oil, or capsules. Indeed, a drug containing curcumin as the active ingredient is now available in the United States as of September 2021.

## 5 CONCLUSIONS

In conclusion, the study confirms that curcumin presents a promising therapeutic target, but most importantly, shows for the first time, the importance of the biological context while delivering it. The order of administration of curcumin and the rhinovirus infection, which may have an important contribution to the development of the remodelling process, significantly affects curcumin effectiveness. These results, although they need to be extended and continued, confirm the usefulness of curcumin in a potential anti‐remodelling therapy and also indicate the pathways of its action that may be useful in understanding the mechanisms limiting the process of airway remodelling in the future.

## CONFLICTS OF INTEREST

The authors confirm that there are no conflicts of interest.

## AUTHOR CONTRIBUTION

**Joanna Wieczfinska:** Conceptualization (lead); Formal analysis (lead); Funding acquisition (lead); Investigation (lead); Supervision (equal); Validation (equal); Writing – original draft (equal). **Przemyslaw Sitarek:** Formal analysis (supporting); Investigation (supporting); Methodology (supporting); Validation (equal); Writing – original draft (supporting). **Tomasz Kowalczyk:** Investigation (supporting); Methodology (supporting); Software (supporting). **Piotr Rieske:** Methodology (supporting); Writing – original draft (supporting). **Rafal Pawliczak:** Conceptualization (supporting); Project administration (supporting); Resources (lead); Supervision (equal); Writing – original draft (supporting).
